# The Neurophysiological Effects of Cervical Transcutaneous Spinal Cord Stimulation With and Without a High Frequency Carrier in Able‐Bodied Adults

**DOI:** 10.1111/aor.15031

**Published:** 2025-06-03

**Authors:** Frances Gawne, Sarah Massey, Lynsey Duffell

**Affiliations:** ^1^ Department of Medical Physics and Biomedical Engineering University College London London UK; ^2^ Aspire Centre for Rehabilitation Engineering and Assistive Technology, UCL Institute of Orthopaedics and Musculoskeletal Sciences Royal National Orthopaedics Hospital London UK

**Keywords:** cervical, high frequency carrier, spinal cord injury, transcutaneous spinal cord stimulation, upper extremity

## Abstract

**Background:**

Transcutaneous spinal cord stimulation (tSCS) is a promising avenue in spinal cord injury (SCI) rehabilitation; however, high currents are required to excite afferents in the spinal cord roots, which patients may not tolerate. Modulating tSCS pulses with a kHz carrier frequency (kHz‐tSCS) may be used to reduce discomfort; however, the way that kHz‐tSCS interacts with neural networks, compared to unmodulated pulses (conv‐tSCS), is largely unknown.

**Method:**

Ten able‐bodied participants received conv‐tSCS, kHz‐tSCS, and sham interventions for 20 min over the C7/T1 vertebrae. Charge delivery of both waveforms was measured. Posterior root reflexes (PRRs) and motor‐evoked potentials (MEPs) were recorded from the Flexor Carpi Radialis (FCR), Extensor Carpi Radialis Longus (ECRL), Flexor Carpi Ulnaris (FCU), and Brachioradialis (BR). PRR and MEP peak‐peak amplitudes were measured at baseline, 0‐, 15‐, and 30‐min post‐intervention.

**Results:**

The charge required to activate posterior roots with kHz‐tSCS was 3.8 times higher than with conv‐tSCS (*p* < 0.001). Differences in PRR amplitude were found in the FCR between conv‐tSCS and kHz‐tSCS at 0‐ and 15‐min post‐intervention (*p* < 0.028). PRR inhibition was found in the FCR between baseline and 30‐min post‐intervention with conv‐tSCS and the sham intervention (*p* < 0.037). No change in PRR amplitudes was found for kHz‐tSCS. No other muscle showed any differences in PRR responses between intervention groups. Neither intervention caused any effect in MEP responses across time or between intervention groups.

**Conclusions:**

kHz‐tSCS was a less efficient waveform for stimulation. Differences in effects on spinal excitability were found to be inconclusive, and conv‐tSCS and kHz‐tSCS had no effect on corticospinal excitability. Significant PRR inhibition in the FCR was found with this experimental setup even when no stimulation was applied, suggesting a natural reduction in spinal excitability caused by participants laying supine for an extended period. Future research should consider how participant positioning could affect neural excitability.

## Graphical abstract

The Neurophysiological Effects of Cervical tSCS With and Without a High Frequency Carrier in Healthy Adults. kHz‐tSCS presents a less efficient waveform. In FCR, spinal inhibition was observed following sham and conv‐tSCS. kHz‐tSCS appeared to prevent FCR spinal inhibition. No effects on corticospinal networks were observed following any intervention.

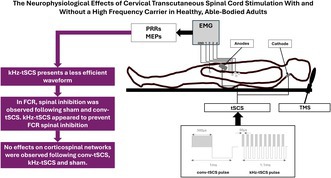

## 1 Introduction

In recent years, pairing physical therapy (PT) with cervical transcutaneous spinal cord stimulation (tSCS) has been a rehabilitation technique investigated to promote upper extremity motor recovery in people with cervical spinal cord injury (SCI) [1, 2, 3, 4, 5]. tSCS aims to non‐invasively stimulate afferents in the posterior spinal cord roots [6, 7], evoking posterior root reflexes (PRRs) via spinal reflex pathways [8]. Delivery of continuous cervical tSCS has been shown to excite upper extremity spinal and corticospinal pathways in people with SCI [1, 3, 9]. As well as this, delivering 20–30 min of continuous cervical tSCS to neurologically intact participants has been shown to induce short‐term neuromodulatory effects on both spinal and corticospinal pathways [10, 11]. A current working hypothesis is that tSCS induces inhibitory effects on spinal networks [12], as well as increasing cortical facilitation [13, 14].

In tSCS, with the stimulation electrodes placed over the skin, relatively high current levels must be used to excite the afferents, compared with epidural spinal cord stimulation [15], which may not be tolerated by some people. Hence, modulating pulses with a carrier frequency in the kHz frequency range has been proposed as a potential method to alleviate discomfort levels of stimulation [16]. Some authors have theorized that delivering pulses of tSCS modulated with a kHz frequency carrier (kHz‐tSCS) in the range of 5‐10 kHz [17] could induce nerve conduction block in pain receptors in the skin [2, 5, 16, 18]. However, in recent years, some authors have suggested that there is no difference in discomfort between kHz‐tSCS and the unmodulated waveform (conv‐tSCS) when pulses are delivered at an intensity that elicits the same PRR amplitude [19, 20]. In addition, kHz‐tSCS has been shown to deliver stimulation less efficiently‐pulses of kHz‐tSCS require higher charge to evoke PRR threshold compared to unmodulated pulses [14, 20, 21]. Some groups have attempted to understand how both waveforms compare in neuromodulatory effects on a healthy nervous system. Research focusing on transcutaneous thoracolumbar stimulation has shown conflicting evidence on how the waveforms compare in facilitating spinal excitation [20, 21]. In our previous trial, we delivered 20 min of continuous stimulation at 40% PRR threshold for each respective waveform [21]. Neither waveform significantly altered H‐reflex threshold, but kHz‐tSCS caused significant PRR inhibition compared to conv‐tSCS, suggesting using the kHz‐tSCS waveform may reduce overactivity in spinal circuitry. In contrast, Dalrymple et al. suggested that the recruitment profiles of both waveforms are similar when delivering single pulses of tSCS at varying amplitudes, implying both waveforms recruit afferents using similar mechanisms [20]. Preliminary evidence from our group suggests that kHz‐tSCS could induce higher corticospinal excitation than conv‐tSCS [21].

With research predominately performed in the context of thoracolumbar stimulation investigating lower extremity neural networks, how both waveforms engage with upper extremity neural networks when delivered to the cervical spinal cord remains largely unclear. Therefore, this study aimed to investigate how delivering cervical tSCS interventions using unmodulated (conv‐tSCS) and modulated (kHz‐tSCS) pulses compares in the short‐term neuromodulation of (i) spinal networks and (ii) corticospinal networks when delivering continuous subthreshold tSCS for 20 min in neurologically intact participants. In addition, this study aimed to compare the charge required for both waveforms to evoke the same level of spinal excitability.

## 2 Methods

### 2.1 Experiment Set‐Up

This experiment was approved by the UCL Research Ethics Committee (Project ID number: 14277/007) and was conducted in accordance with the Declaration of Helsinki. All participants gave written informed consent prior to participating in the study.

The experiment was conducted on 10 able‐bodied participants (mean age ± standard deviation (SD); 25.5 ± 6.49 years; 7 females). The exclusion criteria of the study were: under the age of 18, damaged nervous system, health issues involving their back or upper limbs, and had any of the following: cardiac pacemaker, unhealed upper limb fracture, pregnancy, neurological degenerative diseases, implants or metal in head (excluding dental) or close to electrode site, history of epilepsy, and lacking capacity to provide informed consent.

For the duration of the experiment, participants were asked to lie on a physiotherapy plinth in the supine position. Electromyography (EMG) signals were recorded by placing two adhesive surface electrodes (Ø 24 mm, Covidien, Medtronic, MI, USA) over the muscle bellies of the Flexor Carpi Radialis (FCR), Extensor Carpi Radialis Longus (ECRL), Flexor Carpi Ulnaris (FCU), and Brachioradialis (BR) muscles in the participant's dominant arm (approx. 3 cm apart for each muscle). EMG signals were amplified (×1000) and filtered (2‐10 000 Hz, with a 50 Hz notch) using a D360R amplifier system (Digitimer, Welwyn Garden City, Hertfordshire, UK), digitized at 5000 Hz (Digitimer 1401) and sampled into data acquisition software (Signal v7.07, Cambridge Electronic Design, Cambridge, UK).

**FIGURE 1:**
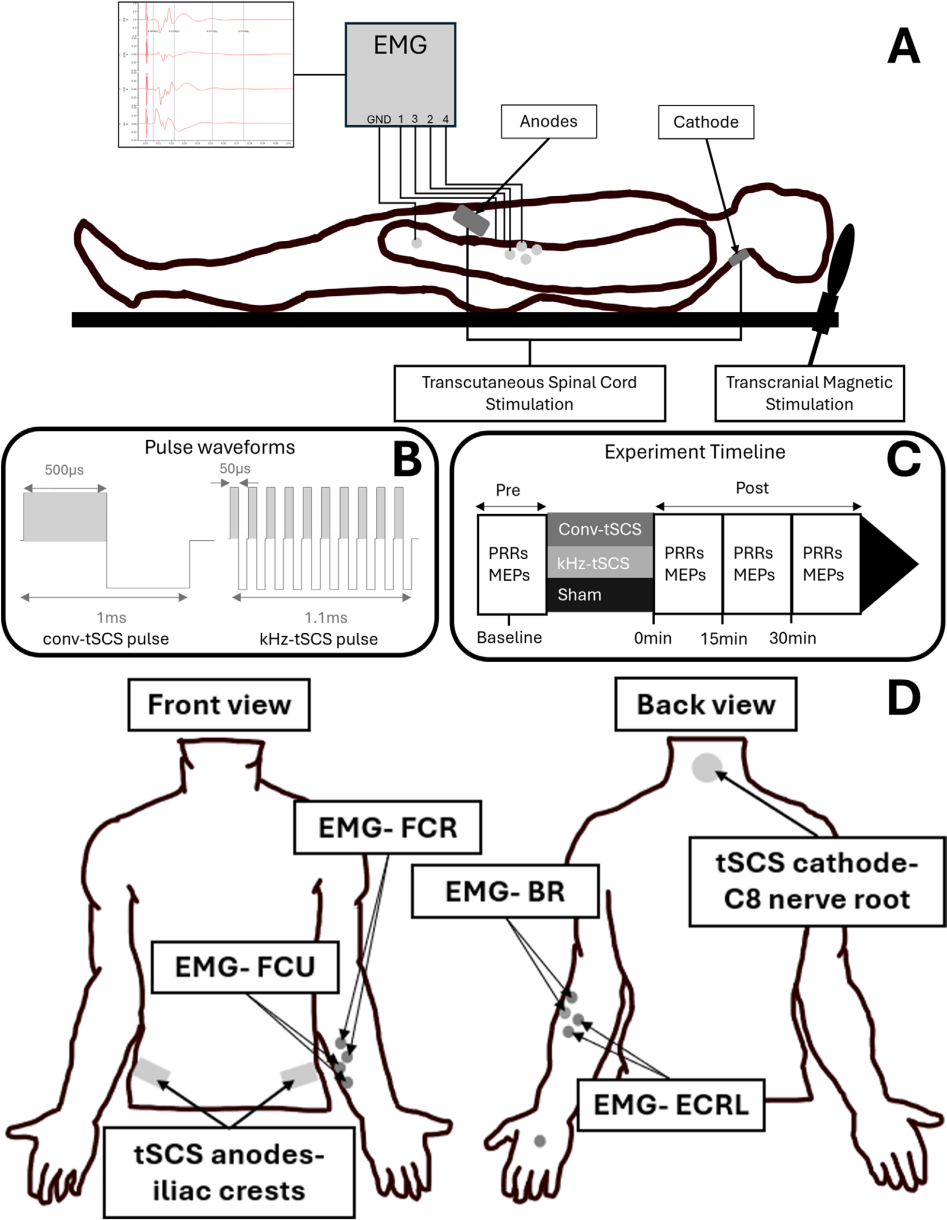
(A) Side‐view of set‐up. (B) Stimulation waveform. (C) Experiment timeline. (D) Location of anodes, cathode and EMG electrodes. BR, brachioradialis; ECRL, extensor carpi radialis longus; EMG, electromyograph; FCR, flexor carpi radialis; FCU, flexor carpi ulnaris; MEP, motor evoked potential; PRR, posterior root reflex; tSCS, transcutaneous spinal cord stimulation. [Color figure can be viewed at wileyonlinelibrary.com]

The anodes (5 cm × 9 cm, PALS Neurostimulation Electrode, Nidd Valley Medical Ltd., UK) and cathode (Ø 5 cm, PALS Neurostimulation Electrode, Nidd Valley Medical Ltd., UK) were placed over the iliac crests and C8 nerve root, respectively (Figure 1).

### 2.2 Experimental Protocol

The participants took part in 3 sessions, at least 24 h apart, each one delivering a different intervention: conv‐tSCS, kHz‐tSCS, or sham. Baseline outcome measures were taken prior to the participants receiving the intervention. The intervention was delivered for 20 min and outcome measures were repeated at 0‐, 15‐, and 30‐min post‐intervention (Figure 1).

### 2.3 Intervention

tSCS was delivered using a constant current stimulator (DS8R, Digitimer, Welwyn Garden City, Hertfordshire, UK), triggered via Signal v7.07 software (Cambridge Electronic Design, Cambridge).

The conv‐tSCS and kHz‐tSCS waveforms are shown in Figure 1B. Conv‐tSCS was delivered using biphasic pulses with a 500 μs pulse width (positive phase) continuously for 20 min at a frequency of 30 Hz. kHz‐tSCS was delivered as bursts of 10 biphasic pulses with a 50 μs pulse width positive phase so that the charge duration matched conv‐tSCS pulses (500 μs positive phase) at a frequency of 9090 Hz. Each burst lasted 1.1 ms, and bursts were delivered continuously for 20 min at a frequency of 30 Hz.

The intensity of both conv‐tSCS and kHz‐tSCS were based on the PRR threshold for each respective waveform. PRR threshold was determined in the FCR using single pulses of conv‐tSCS or single bursts of kHz‐tSCS. PRR threshold was found by increasing stimulation current until the peak‐to‐peak amplitude (p–p amp) of the FCR PRR response reached 0.05 mV. For the 20‐min intervention, conv‐tSCS and kHz‐tSCS were both delivered at 60% PRR threshold or the highest intensity tolerated by the participant, whichever was lower. This intensity was chosen because we have previously shown corticospinal facilitation following a short (10‐burst) 30 Hz train of kHz‐tSCS at ≥ 60% PRR threshold in neurologically intact participants [21] and, in our experience, neurologically intact participants cannot tolerate intensities > 60% PRR threshold over 20 min. Previous studies applying kHz‐tSCS in people with SCI have reported therapeutic effects when applying stimulation at intensities below or at PRR threshold, determined using conv‐tSCS pulses [2, 22, 23]. Therefore, the intensity we applied was comparable to that used therapeutically, at least for the kHz‐tSCS waveform.

The sham intervention provided no stimulation; however, participants were blinded to this. Electrodes were placed, and the threshold was determined in a similar way to the other two interventions, and participants were informed that some interventions were delivered at an intensity that could be felt and others at an intensity below the sensory threshold.

### 2.4 Outcome Measures

#### 2.4.1 Charge Delivery

The current delivered to reach PRR threshold in the FCR with each waveform at baseline was noted. Subsequently, charge delivery, per pulse, at PRR threshold was then calculated using the equation:

Q=It

Where *Q* = charge, *I* = current, and *t* = time of pulse in positive phase.

#### 2.4.2 Posterior Root Reflexes

PRRs were measured to investigate spinal excitability. They were evoked using pulses of conv‐tSCS (as described above). Recruitment curves for each muscle were taken starting at a sub‐threshold (~3 mA below PRR threshold initially measured in the FCR) intensity, increasing to a supramaximal intensity (~1.8 PRR threshold) or when a participant's maximum tolerance was reached, whichever was lower. From the recruitment curves, current required to reach PRR threshold at each time point was measured for each muscle. This was defined as the current intensity at which responses reached 0.05 mV p–p amp, or, if artifact caused the curve to start above 0.05 mV, where the curve started to rise. Prior to the intervention, pairs of pulses were applied at 1.2 × PRR threshold with a 50 ms interstimulus interval. The p–p amplitude of the first and second PRR was measured in the FCR. To document the extent of afferent fiber activation, the p–p amp of the second pulse was expressed relative to the p–p amp of the first PRR. In addition, at each post‐intervention time point, sing pulses were applied at a current intensity equivalent to each muscle's 1.2 × PRR threshold at baseline.

#### 2.4.3 Motor Evoked Potentials

Motor evoked potentials (MEPs) were measured to investigate corticospinal excitability. They were evoked in the upper limbs by transcranial magnetic stimulation (TMS). TMS was delivered over the arm area of the primary motor cortex with a MagStim2002 stimulator (Magstim Co. Ltd., UK) and a circular coil (clockwise current direction for left arm dominant participants, anticlockwise for right arm dominant participants). Participants wore a wig cap that was traced on to keep the positioning of the coil consistent when the optimal position was found for the largest MEP responses.

MEP threshold was determined at the beginning of each experiment (intensity (% maximum stimulator output) that elicited MEPs of 0.05 mV p–p amp in the FCR). At each time point, 10 TMS pulses were delivered at 1.2 × MEP threshold (every 12 s). The p–p amps of the 10 MEPs were averaged.

### 2.5 Data Analysis

All data is presented as mean ± SD, unless stated otherwise. All statistical analysis was performed using SPSS (IBM SPSS Statistics, IBM Inc., version 29.0). Data variables were arranged by time point and intervention (for each measured muscle). Variables were tested for normality using the Shapiro‐Wilks test. If variables did not pass the Shapiro‐Wilks test, all the variables in the dataset were transformed, either using log or square‐root transformations.

A paired t‐test was performed to compare charge delivery between the two waveforms. A repeated measures test was then performed across each intervention condition using the baseline PRR threshold measurements. This was to test for any significant differences between baseline measurements during each condition. If there was no difference, a two‐way ANOVA would be performed using the repeated measures test when variables passed normality testing or the Kruskal Wallis test when variables were not normally distributed. If there were significant differences between intervention groups at baseline, then two one‐way ANOVAs would be performed either using the repeated measures test when variables passed normality testing or Friedman's test when variables were not normally distributed. A one‐way ANOVA would be performed for each intervention group separately using the unnormalized data. Separately, a one‐way ANOVA would compare intervention groups at each post‐intervention time point, where the data would be normalized to baseline. For all statistical testing performed above, the significance value was set to *p* < 0.05.

## 3 Results

Testing baseline PRR threshold values, a significant difference was found between interventions in the ECRL (*p* = 0.003) and BR (*p* = 0.039). Therefore, two sets of one‐way ANOVAs were performed, as described above.

### 3.1 Charge Delivery

Table 1 presents the current and charge required to reach PRR threshold in the FCR at baseline in each participant with both waveforms.

The mean current required to reach PRR threshold was 40.7 ± 11.4 mA with the conv‐tSCS waveform and 150.6 ± 42.9 mA with the kHz‐tSCS waveform. On average, the current required to reach PRR threshold in participants with the kHz‐tSCS waveform was 3.8 times higher than that with the conv‐tSCS waveform. There was a significant difference between the charge delivered with the conv‐tSCS waveform (20.9 ± 5.7μC) and the kHz‐tSCS waveform (76.1 ± 21.6μC; *p* < 0.001).

### 3.2 PRR Amplitude

Pairs of pulses applied at baseline were performed at baseline to assess the extent that afferent or efferent nerve roots were activated by the tSCS. We found that the second PRR was inhibited by 86.6% ± 25.0%, 83.8% ± 26.1%, and 88.7% ± 14.1% of the first PRR p–p amp during the conv‐tSCS, kHz‐tSCS, and sham groups, respectively.

### 3.3 Recruitment Curve

**FIGURE 2:**
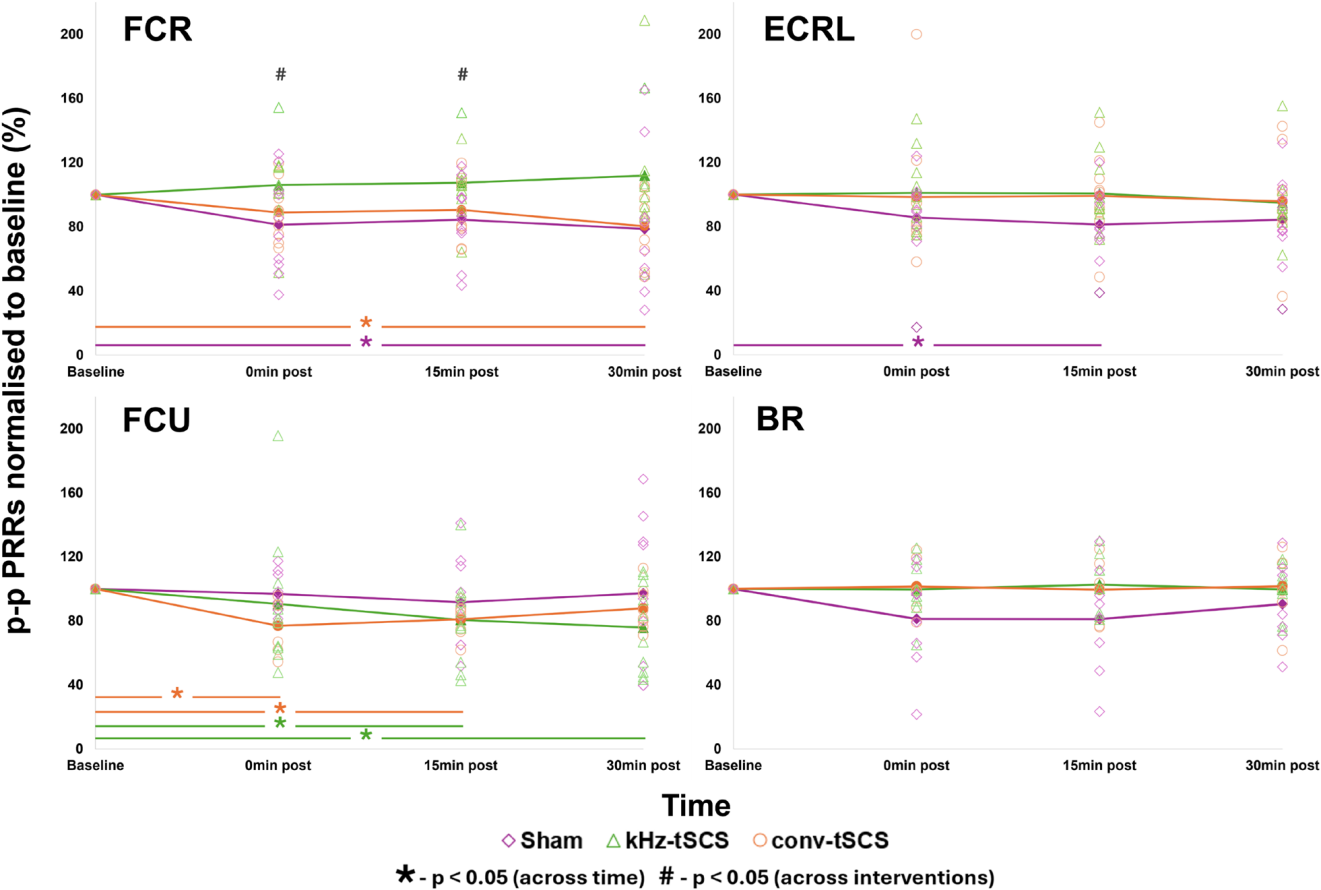
p–p PRR amplitude (measured in the FCR, ECRL, FCU, BR) at 1.2 × PRR threshold normalized to baseline at baseline, 0‐, 15‐, and 30‐mins post‐intervention. Bold lines represent the average p–p PRR amplitude for each intervention. BR, Brachioradialis; conv‐tSCS, transcutaneous spinal cord stimulation with unmodulated pulse; ECRL, Extensor Carpi Radialis Longus; FCR, Flexor Carpi Radialis; FCU, Flexor Carpi Ulnaris; kHz‐tSCS, transcutaneous spinal cord stimulation with pulse modulated with kHz frequency carrier; PRR, posterior root reflex. *p < 0.05 across time. #*p* < 0.05 across intervention groups. [Color figure can be viewed at wileyonlinelibrary.com]

Figure 2 shows PRR p–p amp at 1.2 PRR threshold at each timepoint for all intervention groups in the FCR, ECRL, FCU, and BR.

In the FCR, there was significant PRR inhibition within the conv‐tSCS group between baseline (0.138 ± 0.045 mV) and 30 min post‐intervention (0.11 ± 0.044 mV; *p* = 0.01). Within the sham group, there was also significant inhibition of PRR amplitude between baseline (0.137 ± 0.068 mV) and 30 min post‐intervention (0.119 ± 0.145 mV; *p* = 0.037). The kHz‐tSCS group did not show any significant changes in PRR response across time. Across interventions, at 0 min post‐intervention there was a significant difference between PRR responses for the conv‐tSCS group (88.9% ± 17.4%) and kHz‐tSCS group (106.1 ± 25.8%; *p* = 0.015). Significant differences between these two groups were also found at 15 min post‐intervention (conv‐tSCS: 90.6 ± 19.2%, kHz‐tSCS: 107.5 ± 23.6%; *p* = 0.028). No other differences were found between intervention groups.

In the ECRL, PRR responses in the sham group were significantly inhibited from baseline (0.125 ± 0.06 mV) to 15 min post‐intervention (0.096 ± 0.036 mV; *p* = 0.036). The conv‐tSCS and kHz‐tSCS groups showed no changes in PRR response across time. There was no statistically significant difference in ECRL PRR amplitude between interventions at any time point.

FCU PRR inhibition was observed in the conv‐tSCS group between baseline (0.234 ± 0.126 mV) and 0 min post‐intervention (0.17 ± 0.081 mV; *p* = 0.016), as well as between baseline and 15 min post‐intervention (0.188 ± 0.105 mV; *p* = 0.005). The kHz‐tSCS group showed inhibition in PRR amplitude between baseline (0.21 ± 0.195 mV) and 15 min post‐intervention (0.168 ± 0.16 mV; *p* = 0.046), as well as baseline and 30 min (0.158 ± 0.168 mV; *p* = 0.029). There was no change in PRR response across time for the sham group. There were no significant differences between interventions.

In the BR, there were no significant changes in PRR responses across time. Across intervention groups, no significant difference was found.

### 3.4 Current Delivered at PRR Threshold

**FIGURE 3:**
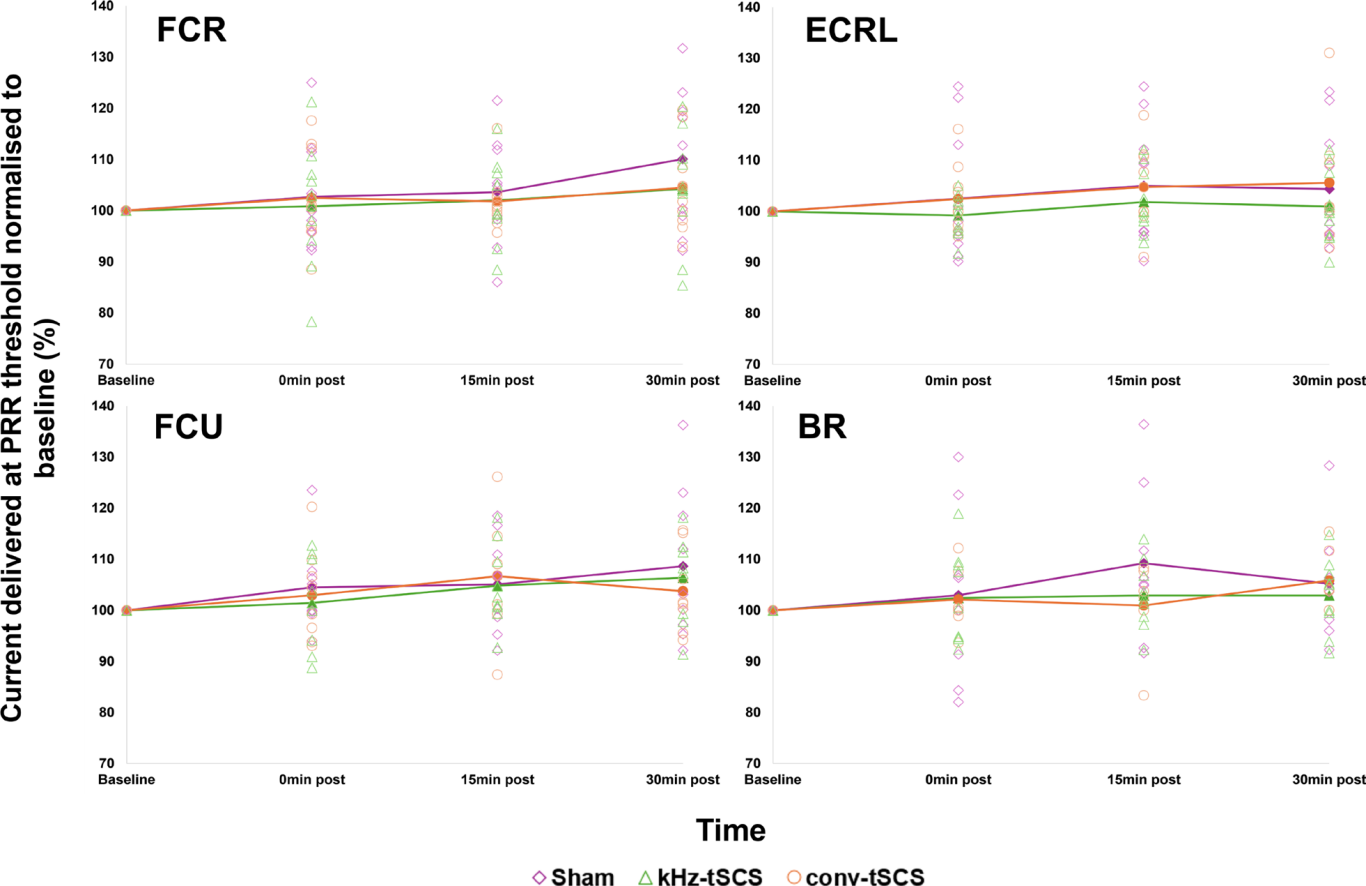
PRR threshold (measured in the FCR, ECRL, FCU, BR) normalized to baseline at baseline, 0‐, 15‐, and 30‐mins post‐intervention. Bold lines represent the average PRR threshold for each intervention. BR, Brachioradialis; conv‐tSCS, transcutaneous spinal cord stimulation with unmodulated pulse; ECRL, Extensor Carpi Radialis Longus; FCR, Flexor Carpi Radialis; FCU, Flexor Carpi Ulnaris; kHz‐tSCS, transcutaneous spinal cord stimulation with pulse modulated with kHz frequency carrier; PRR, posterior root reflex. [Color figure can be viewed at wileyonlinelibrary.com]

Figure 3 shows the current intensity required to elicit PRR threshold at each timepoint across interventions, in the FCR, ECRL, FCU, and BR.

There were no statistically significant changes to the current required to elicit PRR threshold for any intervention group in any muscle.

### 3.5 MEP Amplitude

**FIGURE 4:**
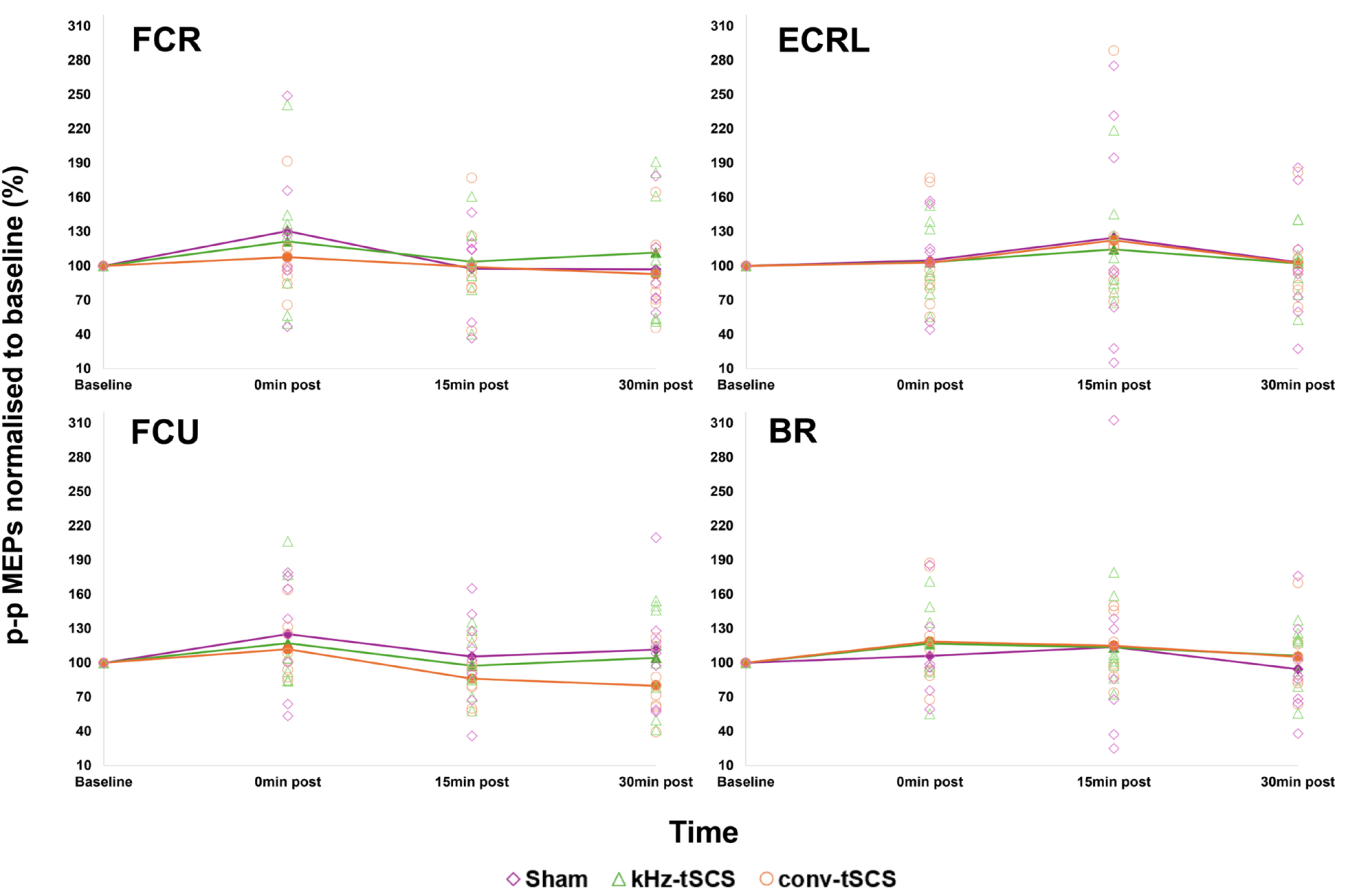
p–p MEP amplitude (measured in the FCR, ECRL, FCU, BR) normalized to baseline at baseline, 0‐, 15‐, and 30‐mins post‐intervention. Bold lines represent the average p–p MEP amplitude for each intervention. BR, Brachioradialis; conv‐tSCS, transcutaneous spinal cord stimulation with unmodulated pulse; ECRL, Extensor Carpi Radialis Longus; FCR, Flexor Carpi Radialis; FCU, Flexor Carpi Ulnaris; kHz‐tSCS, transcutaneous spinal cord stimulation with pulse modulated with kHz frequency carrier; MEP, motor evoked potential. [Color figure can be viewed at wileyonlinelibrary.com]

Changes in p–p amp of MEP responses over time, for different intervention groups, in the FCR, ECRL, FCU, and BR are shown in Figure 4.

In all muscles measured, no significant change in MEP amplitude was found across time for all interventions. No significant differences between intervention groups were found in any muscle.

## 4 Discussion

The present study compared 20 min of conv‐tSCS, kHz‐tSCS, and a sham intervention on spinal (PRR amplitude) and corticospinal (MEP amplitude) excitability in upper limb muscles of able‐bodied participants. Our main finding was that conv‐tSCS and the sham intervention caused significant spinal inhibition in the FCR, which was not evident when kHz‐tSCS was delivered. Mixed results were found in the other muscles measured.

### 4.1 Spinal Excitability

In the FCR, both the conv‐tSCS and sham interventions caused inhibition in PRR responses, whereas the kHz‐tSCS intervention did not, which indicates that kHz‐tSCS may facilitate PRRs. This finding contradicts our previous trial, which found that kHz‐tSCS caused significant inhibitory effects on PRRs that were not present with conv‐tSCS [21]. This previous trial was conducted with thoracolumbar rather than cervical tSCS; therefore, the effects of conv‐tSCS and kHz‐tSCS appear to differ between upper and lower limb muscles. Thoracolumbar stimulation causes a greater amount of post‐activation depression (second response amplitude < 10% of first [8]) than we have found in the upper limb muscle; it is possible that the relative activation of afferent fibers differed between the two waveforms during cervical tSCS. In addition, there were differences in intensity used between this trial (60% PRR threshold) and our previous trial (40% PRR threshold) that could have contributed to the differences in effects found. One previous trial conducted using cervical tSCS found that conv‐tSCS had no statistically significant effect on spinal excitability in upper limb muscles [24]. This is similar to our result found in the FCR, where conv‐tSCS had no effect on spinal excitability when compared to the sham intervention. Our results indicated that FCR spinal inhibition can be caused by 20 min of lying supine (as shown by sham intervention results) and applying conv‐tSCS had no effect on this. It should be noted, however, that Sasaki et al. delivered conv‐tSCS in a supine position but for 10 min (compared to 20 min in our trial). They also suggested that the stimulation intensity they set, which was the intensity to induce paraesthesia, may have been too low for conv‐tSCS to fully engage with spinal networks [24].

In our current trial, significantly more charge was delivered during the kHz‐tSCS intervention as a higher charge was required to reach PRR threshold in the FCR compared to the conv‐tSCS intervention. As the current waveform of the kHz‐tSCS intervention is a high frequency pulse burst, it relies on temporal summation to trigger CAPs in the afferents [25, 26]. During the positive phases, the membrane potential of the afferents' axons increases closer to AP threshold [27]. However, during the negative phases in the pulse burst, the membrane potential will slightly decrease, effectively wasting current that actively raised the membrane potential toward AP threshold. Therefore, it is often considered that kHz‐tSCS delivers less efficient stimulation over conv‐tSCS [14, 20, 21].

In the current trial, the effects of each intervention on spinal excitability differed between the upper limb muscles measured. In the ECRL, the sham intervention caused PRR inhibition, whereas the conv‐tSCS and kHz‐tSCS caused no change in PRR amplitude. In the FCU, conv‐tSCS and kHz‐tSCS caused PRR inhibition, whereas the sham intervention caused no changes in PRR amplitude. It should be emphasized that intervention intensity was set based on the PRR threshold value found in the FCR. As can be seen from Table 1, the average PRR threshold found in the other measured muscles was similar to that found in the FCR. However, a factor in the discrepancies in effects on spinal excitability found in each muscle could be due to inconsistency in the stimulation intensity relative to threshold across different nerve roots (Table 1). The ECRL and BR are both innervated by the radial nerve [28] and showed similar patterns of spinal excitability between interventions (Figure 2). The FCR and the FCU, innervated by the median nerve and the ulnar nerve, respectively [28], showed different patterns of spinal excitability. It should also be acknowledged that the placement of the cathode over the back of the neck may have caused activation of the peripheral nerves of more cranial segments, at least in some participants. This may have contributed to the variability in our results.

**TABLE 1:** Current required to elicit PRR threshold in the FCR, ECRL, FCU, and BR, and the intervention current and charge delivered with the conv‐tSCS and kHz‐tSCS waveforms for each participant.

Muscle	PRR threshold (mA)					Intervention intensity (mA)		Intervention charge (μC)	
	FCR		ECRL	FCU	BR				
Waveform	conv	kHz	conv	conv	conv	conv	kHz	conv	kHz
Participant									
P01	54.0	93.0	45.7	38.0	49.0	32.4	55.8	16.2	27.9
P02	28.0	93.0	21.5	23.7	31.0	16.8	55.8	8.4	27.9
P03	42.0	117.0	41.6	37.0	39.5	25.2	70.2	12.6	35.1
P04^a^	34.4	142.0	30.7	33.0	34.0	11.0	45.4	5.5	22.7
P05	33.5	178.0	38.0	37.9	41.2	20.1	106.8	10.1	53.4
P06	63.5	200.0	59.0	56.4	59.0	38.1	120.0	19.1	60
P07	42.5	185.5	53.1	36.5	34.8	25.5	111.3	12.8	55.7
P08	45.0	200.0	64.1	58.4	46.2	27.0	120.0	13.5	60
P09	33.0	175.0	31.9	31.6	34.1	19.8	105.0	9.9	52.5
P10	32.5	147.2	25.0	25.7	26.7	19.5	88.3	9.8	44.2
Ave ± SD	40.7 ± 11.4	150.6 ± 42.9	41.1 ± 14.4	37.8 ± 11.4	39.6 ± 9.6	23.5 ± 7.8	87.9 ± 28.7	11.8 ± 3.9	43.9 ± 14.4

Abbreviations: Ave, average; BR, brachioradialis; ECRL, extensor carpi radialis longus; FCR, flexor carpi radialis; FCU, flexor carpi ulnaris; PRR, posterior root reflex; SD, standard deviation; tSCS, transcutaneous spinal cord stimulation.

^a^ Participant P04 received conv‐tSCS and kHz‐tSCS at 32% PRR threshold as this was their maximum tolerable intensity.

### 4.2 Corticospinal Excitability

From this investigation no intervention caused any changes in MEP amplitude across time, nor were there any significant differences in MEP amplitude between the three interventions. In agreement, Sasaki et al. found that conv‐tSCS had no effect on corticospinal excitability in upper limb muscles, but they did not investigate kHz‐tSCS [24]. Again, these authors suggested this result could be due to a low stimulation intensity.

There is a contradiction in the current literature on how conv‐tSCS and kHz‐tSCS compare in terms of corticospinal excitability. Our previous study suggests that kHz‐tSCS may cause short‐term corticospinal facilitation to a greater extent than conv‐tSCS [21]. However, Benavides et al. suggest the opposite: that in neurologically intact participants, kHz‐tSCS intervention has no effect on corticospinal pathways, while conv‐tSCS did [10]. It should be noted that they delivered cervical tSCS, unlike our previous study, again alluding to the potential that the effects of tSCS may differ between upper and lower limbs. Indeed, corticospinal facilitation by afferent stimulation in lower limb muscles has been reported to be non‐specific and diffuse [29] whereas facilitation in the upper limb appears to be more muscle‐specific [30, 31]. Additionally, the greater corticospinal effects caused by kHz‐tSCS from our previous study could be a result of larger current spread due to this waveform, causing more afferents to be recruited [21].

### 4.3 The Justifications for High Frequency Carriers

As mentioned previously, the potential that kHz frequency carriers may induce conduction block on pain receptors in the skin is one theory to justify their use within tSCS. However, an aspect that may have been overlooked is that this theory is based upon applying a continuous high frequency alternating current (HFAC) directly to an animal nerve [32]. It is not certain that this theory still holds when stimulation is applied transcutaneously, in short bursts with a square waveform. Additionally, some evidence suggests there is an ‘onset’ period of neural activity when applying a HFAC to a nerve, before the conduction block is induced, that lasts ~50‐100 ms [33]. With pulse widths lasting at most a few milliseconds for tSCS application [15], a full block may not have time to be induced. Manson et al. and Dalrymple et al. both quantified that when delivering tSCS with both waveforms to the same level of neural engagement, participants experienced no difference in discomfort levels of the stimulation [19, 20]. Another justification of the use of high frequency pulse modulation is that this waveform could potentially reduce the impedance of cutaneous tissue, leading to a higher potential reaching the posterior root afferents [16]. However, again this theory was based on HFAC, and with the short duration of pulse bursts used in this experiment, this is unlikely to be the case.

### 4.4 Limitations and Future Work

This experiment was performed with a small sample size and a larger participant pool was needed to increase the reliability of the results.

It is possible that the intensity of the cervical tSCS interventions was not high enough to fully engage with neural networks in able‐bodied participants. Previous studies have also considered this concept [21, 24]. This study chose to stimulate at 60% of a participant's PPR threshold in the FCR, as our previous study found that pulse trains at this intensity were able to elicit corticospinal excitation [21]; however, from previous experience, neurologically intact participants cannot tolerate 20 min of continuous stimulation at a higher intensity. It should also be acknowledged that the effects of tSCS may be less evident in people without neurological impairment [24]. Further investigation should explore similar or higher stimulation intensities in people with SCI. In addition, the inclusion of physical training should be considered in future trials, as this has been found to facilitate neural engagement during a cervical tSCS intervention in neurologically intact participants [11, 24].

Finally, this study aimed to help understand how to optimize cervical tSCS parameters to best facilitate upper body rehabilitation to people with cervical SCI. Hence, it is imperative to understand the differences in neurophysiological effects that conv‐tSCS and kHz‐tSCS elicit on an injured nervous system.

## 5 Conclusion

This study found that spinal inhibition in the FCR muscle was prevented when kHz‐tSCS was applied. However, no conclusive difference between how conv‐tSCS and kHz‐tSCS affects spinal excitability was found. It should be emphasized that spinal inhibition was caused by 20 min of lying supine without continuous stimulation. No differences were found between conv‐tSCS and kHz‐tSCS in terms of corticospinal excitability in able‐bodied participants. Further investigation is required to confirm this conclusion. The inclusion of a carrier frequency in the tSCS waveform may decrease the efficiency of stimulation. Further research is required to determine the benefit of incorporating a kHz carrier frequency in cervical tSCS.

## Author Contributions

Lynsey Duffell and Sarah Massey designed the investigation; Frances Gawne performed the investigation; Frances Gawne performed the data analysis; All authors interpreted results; Frances Gawne prepared the figures; Frances Gawne wrote the manuscript; All authors reviewed and approved the final version of the manuscript.

## Conflicts of Interest

The authors declare no conflicts of interest.
